# A conceptual framework for analysis of environmental security and development based on source and flow concepts

**DOI:** 10.1038/s41598-025-99532-5

**Published:** 2025-04-29

**Authors:** Tianhai Zhang

**Affiliations:** 1 Key Laboratory of Land Resources Evaluation and Monitoring in Southwest (Sichuan Normal University), Ministry of Education, Chengdu, 610066 China; 2 Department of Arts, Science, and Technology, Sichuan Normal University, Chengdu, 610101 China

**Keywords:** Environmental security and development, Cell model, Source-access-flow, Environmental impact, Ecology, Ecology, Environmental sciences, Environmental social sciences, Risk factors

## Abstract

Researches on security and development (SD) is interrelated and essential for ecosystem especially human survival but commonly separated and diverged to multiple scales and fields. Thus, a more integral and fundamental theoretical framework is required for following better analysis of both of the dual concepts, like the United Nations Sustainable Development Goals need the more innovative interdisciplinary theoretical framework to face comprehensive challenges of both sustainable development and security in nowadays. Therefore, this research aims to integrate the study of the two aspects and proposes an abstract, general fundamental framework for analysis of environmental impact on them. Based on theory of Environmental Economic Geography (EEG), Ecosystem Services (ES) and concepts of ‘Source-Access-Flow’ (SAF), ‘Cell model’ with its ‘Cell Compass’ approach for analysis of environmental spatial pattern and corresponding strategies was established for such framework. The analysis results of Cell model classified and ranked different typical environmental patterns as well as corresponding actions, so as to address environmental pattern assessment and optimize strategies for better development situation. Further, possible changes were discussed and calculation methods of development level and efficiency were defined.

## Introduction

This research mainly focuses on discussion of the environmental impact on the security and development of a system ^1,2^. Thus, it’s a theory of environmental security and development (ESD).

In this topic, firstly the description of the concept of ‘system’ should be defined but it is complex as it could refer to both organic and inorganic systems. For this study, it mainly refers to organic systems (OS) involves different levels and scales from individual to complex huge ecosystem. Taking human society as an example, it could differ from communities, cities, regional areas, and even countries ^1–3^.

The second couple of concepts should be defined and clarified is the ‘security and development’. Separately and originally, ‘development’ was a biological concept referring to the growth process of biological individuals from small to large, from immature to mature ^4,5^. However, current research of development science broadened from reductionism focusing on biology, psychology, and sociology of individual (like human development) to sort of paradigm based on much more complex and interacting systems ^6–8^.

Further, the another interdisciplinary field of development study, originated from economics but mixed with concepts of growth ^9–11^, extended to many fields such as sociology, philosophy, political science and history and became an inter- and multi-disciplinary subject, covering various social and scientific fields^1^.

Meanwhile, inevitably, for development studies, its relationship with human security research provided another example of its interdisciplinary nature. The emergence of human security—a new, people-oriented approach to understanding and responding to global security threats—has led to a growing awareness of the relationship between security and development ^12^. Human security believes that inequality and insecurity in a country or region will have an impact on global security, so it is in the interests of all countries to solve fundamental development issues. It is therefore that the discussion of ‘development’ issue in this research highly related to the issue of ‘security’ ^12–14^.

Therefore, ‘security and development’ is a couple of concepts in this research rather than separate ones, considering that it should refer to ‘development’ when the environmental factors is beneficial, and it would shift to ‘security’ when the environmental factors is deleterious. Therefore, the concepts of security and development are closely linked dual-concepts to form the integrity of this research.

For research of security and development, which factors affect them are important topics. Generally, these factors could be divided into two main categories, internal factors and external factors (environmental factors). While this research mainly focused on the external factors (environmental factors), ignoring the internal factors, and try to set up analysis framework of how environmental impact on security and development based on the concepts of environmental source and flow (ESF).

For environmental impact on security and development at different scales, related discipline differ from environmental psychology, ecosystem development to environmental ecology, environmental sociology, urban metabolism, sustainable development, etc. ^15,16^. Takes environmental psychology as an example, it emphasizes on how humans change the environment and how the environment changes humans’ experiences and behaviors. That means it explores the interactive relationship between humans and the external world ^17^.

Environmental sociology focused on the expansion of early sociology by incorporating the physical environment related to social factors ^18,19^. Emerged as a sub-field of sociology in the late 1970s, environmental sociology is a discipline that studies the interaction and relationship between society and its natural environment. The interaction between human beings and the natural environment differs in many aspects, such as health and disease, population and demography, consumption and sustainable practices.

Similarly, urban metabolism provides researchers with a metaphorical framework to study the interactions of natural and human systems in specific regions ^20,21^. Sustainable development aims at achieving the goal of human development without destroying the integrity of the earth and the stability of natural resource systems ^22^. Put simply, it focused on balance of economic development, social development and environmental protection for future generations ^23–25^.

For exploring the influencing factors of economy since 1950s, Economic geography (EG) gradually introduces ‘environmental’ factors and pleas for an ‘environmental economic geography ‘(EEG) ^2,26,27^. EEG presents the relationship between economy and environment, like how the economic activities change the environment and how the environmental base generates impact on economic activities. Further, the EEG also studies the relationship between the patterns of economic development and spatial layout of resources in environment, as well as the adaption to changes ^2,28,29^. Meanwhile, spatial Environmental economics was formed to join the spatial concern of interaction between economy development and environment and examined their mechanism well from the perspective of geographical space ^28,38^.

The ecosystem services (ES) theory is another highly related research field for regional sustainable development. Since ES was set up in 1990s by Daily and Costanza et al. ^30,31^, many scholars have made numerous initiatives, efforts and results on its two main contents, the Supply and Demand, as well as its balance or mismatches, including more integrated, interdisciplinary approaches, considering not just the economic value but also the social and cultural benefits ecosystems provide^32–34^. Among them, Bakshi proposed a framework of Techno-Ecological Synergy (TES) for connection of ES demand and ES supply at multiple spatial scales, combining many existing methods^35–37^.

As mentioned before, the security issue is closely interrelated with development issue. While, current researches or practices like the urban planning tends to address such issues in a separate manner instead of integrated ^39,40^, see the EEG theory mainly focused on the economy development. However, The United Nations Sustainable Development Goals (SDGs) try to guide global development efforts to focus on more attention on security issues like energy cleanliness and security, social well-being, equity and justice ^41–43^. Thus, nowadays the hot topics and key research issue of the security like food security, energy security, water security, or other ecology security which would affect the sustainable development for human survival lack attention in the EEG and require evolutionary theoretical framework ^44–46^. That means the realization of SDGs in the future would provide new requirements and opportunities to development and update the EEG based on integration of multi-perspectives and interdisciplinary.

Similar situation for ES, as the Millennium Ecosystem Assessment defined in 2005, the ES emphasized the importance of human well-being and the benefits that humans obtain from ecosystems ^47^. However, a gap with interdisciplinary integration exists due to that the ES didn’t include the content of potential hazards, threaten and risks from the environment, such as the crisis of food, energy, or water, carbon footprint, climate disasters, and other security issues beyond development emphasized by the United Nations SDGs ^41–43^. Generally, the ES research takes such threaten and risks as external factors that would affect ES. But this research attempts to establish a framework with its model and approaches to integrate both services and threaten as the external environment or background of a system, thereby proposing a theory to focus on the environmental impact on system security and development, namely ‘environmental security and development (ESD)’.

Specifically, the ESD employs two separate concepts of ‘source’ and ‘flow’ in ecology, combined them together and extended them to ‘source-access-flow’ (SAF) to describe and analyze the environmental impacts on security and development. Thus, the ‘source-flow’ is also named ‘environmental source and flow (ESF)’ in this research and covers all aspects of the environmental base including natural and human-made flow that would affect security and development ^1^, not just about water flow as commonly defined ^48,49^.

Originally, the concepts of source–sink dynamics are used by ecologists to describe how habitat quality might affect the population growth or decline of organisms ^50–52^. For social production activities in industrial system, ecologists regard it as an entire ecosystem and study the cycle of natural resources with concepts of “source”, “flow” to “sink” ^53^. While concept of environmental flow aim to allocate sufficient water to ecosystems to maintain a certain level of ecological integrity based on an appropriate management vision. Similar terms like ‘ecological flows’, ‘instream flows’, ‘minimum flows’, and ‘normative flows’ convey much the same concept ^54–57^. Then ‘environmental flow’ extensively accommodates all components of the river including natural flow variability, socioeconomic, and ecological aspects but causes broad confusion in wide use ^54,58^.

The flow in ES theory is also an essential concept because it relates the ES supply and ES demand and clarifying the specific flow paths of ES help analyzing the dynamic balance of ES supply and demand, providing scientific basis for policies making and achieving the goal of sustainable development ^35,59^. However, studies on the ES flow currently are still in the preliminary exploration stage, and quantitative evaluation are not yet systematic ^60,61^, as well as lack of considerations of service demand.

Based on source-access-flow concepts and the review of related theory above, like EEG try to answer how the environmental base and economic activities impact each other, this research aim to set up an abstract and fundamental theoretical framework of ESD about how the ESF affect the security and development. Unlike the ES flow focus on the benefit process from the supply area to the beneficiary area ^62^, this research analyzes both beneficial and deleterious flow. In addition, ES synthesizing its supply and demand is essential so as to couple nature and humans, finally achieving harmony between human activities and nature. While, this research not only consider the nature factors but also the human activities in the background for system security and development. The natural factors and human-made results that serve as the background of the system are collectively referred to as the environment in this research, and are reclassified as beneficial and deleterious sources.

Thus, the proposed framework in this research tries to synthesize the security and development for a system when facing environmental influence. Especially, with concepts of SAF in this framework, the linkage of the issues of security and development become more interrelated, that means when the flow is beneficial, it’s more about development and service, when the flow is deleterious, it’s more about threaten, risk and security. Further, this research also discussed the corresponding strategies from the system when it face the environment, as well as the possible situations, patterns, and consequences due to the mutual influence between system and external environment under a specific ‘Cell Compass’ named analysis approach in ‘Cell model’.

Like the TES framework using many existing methods at multi spatial scales ^35^, this research across disciplines combined the advantages of various disciplinary methods like ecology, geography, and even economics at multiple scales ranging from an individual process to regional local or even global. The characteristics of this framework is that, each concept is generalized, abstracted and meaningful to make the framework more integral, fundamental but meanwhile bringing a contradiction that the discussion appears vague, unclear, and non-specific. Therefore, in the following discussion of ESD, for better understanding, a selected scale and specific object of urban ecosystem could be taken into the discussion of an organic system in this study considering that the meaning of each single concept are very complex.

## Materials and methods

### Cell model

#### Hypothesis and interpretation

The hypothesis for Cell model could be introduced in three concise sentences:

1. There’s a system and its external environment;
2. Ignoring the intrinsic attributes and internal factors, the system would increase when gaining beneficial flow from external environment, and decrease when gaining deleterious flow;
3. When there’s no beneficial flow or deleterious flow, the system is supposed to stay unchanged.

**Fig. 1:**
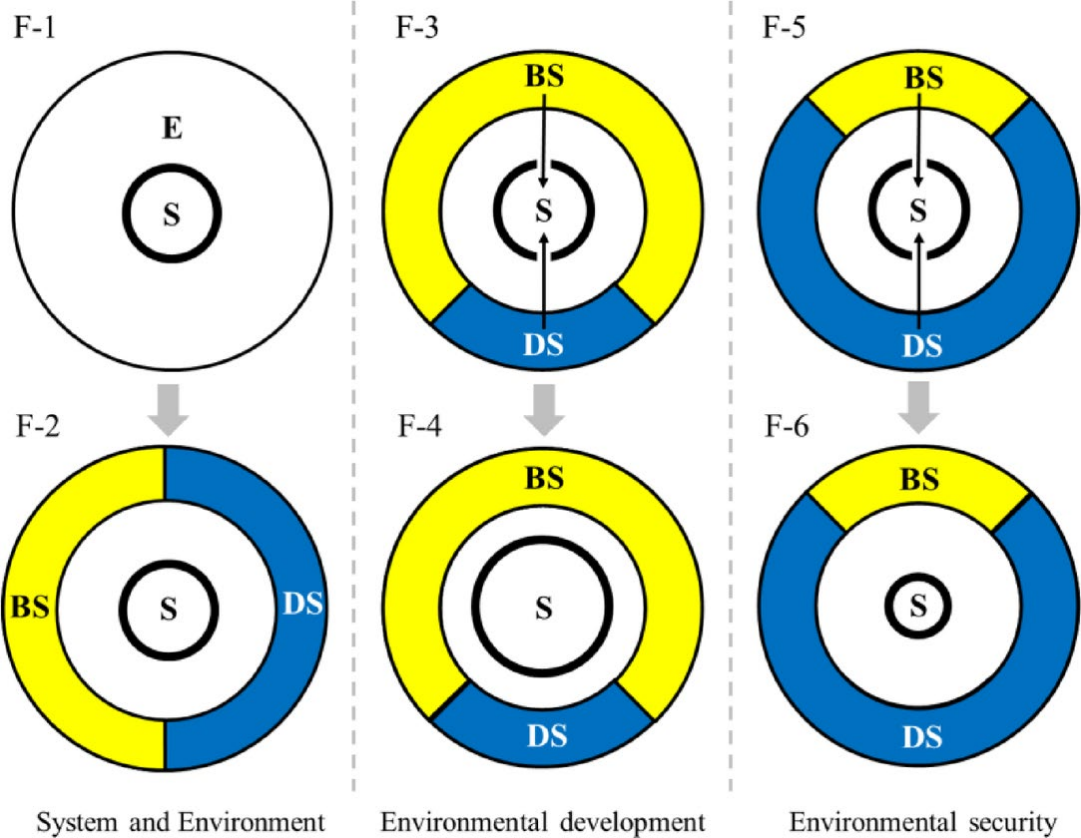
The description of cell model (E = environment, S = system, BS = beneficial source, DS = deleterious source, meanwhile, blue represent the deleterious source and yellow represent the beneficial source without more explanation in the later figures).

The following Fig. 1 described the Cell model:

There are 6 sub-figures in Fig. 1, the F-1 on the left side presents the relationship between the system and the external environment. For better understanding of environment impact on system development and security by various factors, the overall environment could be further subdivided into beneficial sources and deleterious sources, as shown in F-2.

Generally, when the system obtain more beneficial flow than deleterious flow from environmental sources by building access, this is mainly a development situation (F-3 → F-4), just like cells absorb nutrients from the surrounding environment and keep growth. However, from a safety perspective, just as the ingestion of environmental deleterious substances by cells would harmfully affect their own development, the intake of deleterious flow from environmental sources by system would be detrimental to its development (F-5 → F-6), resulting in security problems. It is therefore that the relationship between the environment and the system always related to both development and security. Development and security, these two concepts are just two opposite perspectives when the system facing one environment.

Again, as shown in F-3 in Fig. 1, when there are more beneficial sources than deleterious sources in the surrounding environment, system could gains more beneficial flows than deleterious flows and finally lead to development (F-4 in Fig. 1); whereas, when system gains more deleterious flows than beneficial flows, it decrease with security problems (F-5 → F-6).

What if there are neither beneficial flows nor deleterious flows? This paper assumes that the system would not be affected (or remains unchanged). For this assumption, a controversial point is that from the metabolic perspective, lack of basic flow necessary for development would lead to system recession. However, for non-basic flow, such lack would cause almost no influence to the system development. While, the classification of basic and non-basic needs highly depend on system intrinsic attributes, which will lead to much more complex discussions. Therefore, since the hypothesis for Cell model has declared no considering of intrinsic attributes of system, this point here is simply processed as ‘no effect’ (or remain unchanged).

The Fig. 1 above is a brief explanation of the assumptions of Cell model, and the following sections are introduction of related core aspects.

#### Source

The concept of ‘Source’ in this paper includes two basic attributes:

Beneficial or deleterious? In source-sink model, Pulliam defines the sources as a net exporter of individuals ^51^. While, this paper defines various factors in the environment as sources. Depend on system special needs or intrinsic attributes, these factors were classified to beneficial sources and deleterious sources (F-2 in Fig. 1). For better description in this research, like the term ‘resource’, the ‘beneficial source’ is named as ‘Be-source’ and the ‘deleterious source’ was named as ‘De-source’ for short.

Static or Dynamic? The significance of discussing the dynamic and static attributes of the source lies in a question that whether a source has to produce a flow? Static sources would not automatically generate flows, while dynamic sources do. Thus, such difference obviously affects the approaches of the flow establishment. Further, such difference would affect the efficiency of system investment and development as well.

#### Flow

As corresponding to the two attributes of the source, the flow also has two key attributes in this study:

1. Be-flow and de-flow: be-flow is short of beneficial flow and de-flow is short of deleterious flow. Be-flow comes from be-source and de-flow comes from de-source.
2. Active flow and passive flow, as discussed in the source section, the attribute difference of static or dynamic would affect the approaches of flow establishment. That means, when the source is static, it requires system to actively build the flow to obtain it. Such building activity would bring investment and cost. For example, the growth of urban development depends partly on fossil fuel, which is an approach for system to obtain energy flows. Such simple example indicates that energy obtaining requires construction efforts and energy input. Whereas, when the source is dynamic, system no longer need to actively construct to obtain the flow, but only need to passively accept it, like the rain for the water flow. Therefore, the static and dynamic attributes of the source and flow affect the input and cost of system, and thus affect their development efficiency.

When considering only there is a flow or no flow for the final result, and ignoring of the specific establishment approach of the flow (actively established flow or a passively accepted flow), the relationship between the system development and the source-flow in the environment in Fig. 1 can be processed as the following matrix table Table 1.

In the matrix table above, there is an interesting situation: why there’s a result with deleterious flow for system development? This question could also be asked as why doesn’t the system prevent the deleterious flow? The possible reason for this could be: misunderstanding, or the flow is out of system control (such as climate).

## Results

### Environment pattern

**Fig. 2:**
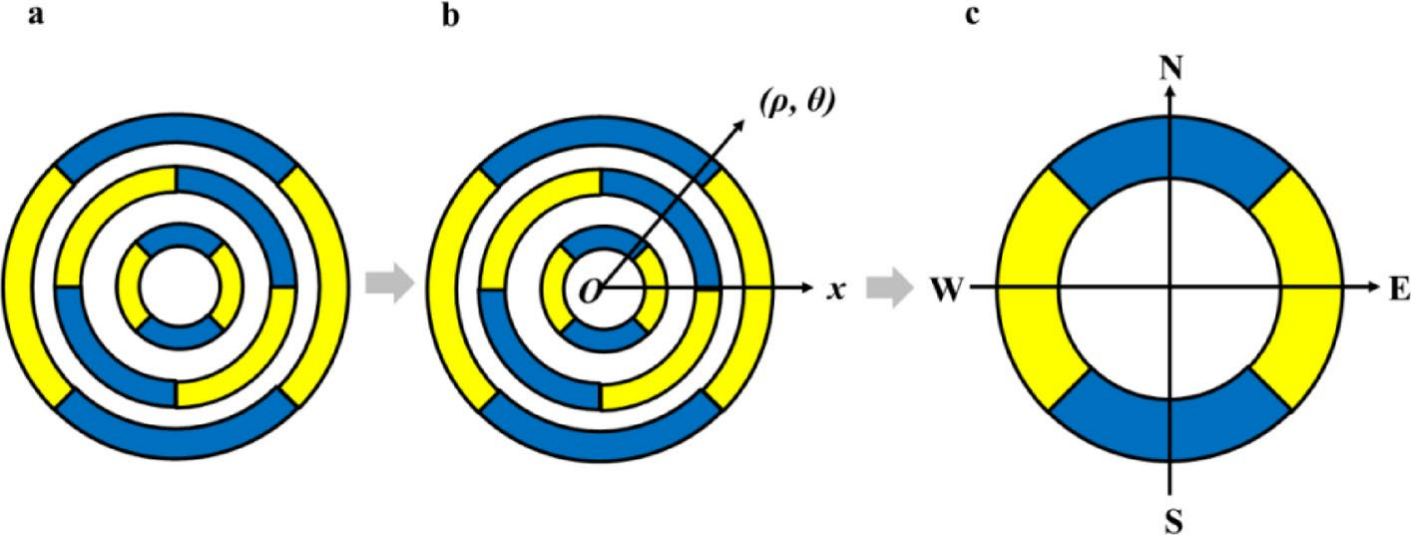
The description of spatial distribution of sources (in part c, E = East, S = South, W = West, N = North).

Based on the introduction of the core factors of the cell model above, this section describes the distribution pattern of the sources in the environment, as shown in Fig. 2.

In general, the distribution of various sources in the real environment may be much more complex than a three-dimensional spatial distribution. Take information sources as example, it would be difficult to describe the spatial location or distribution. It is therefore that always the descriptions of the real complex world are tough work. Notably, such fully considering all complex factors in the real world is not conducive to clear the basic discussions and analysis for establishment of new fundamental theories. Therefore, for general fundamental theory and its framework of ESD, this paper just tries to simplify the description of the distribution complexity by using sort of spatially visualized planar representations. That means, firstly, we would clear the simplest situations and cases, and then gradually broadened to complex cases. Such simplified description of spatially visualized planar representations is shown in the following Fig. 2.

In Fig. 2, part a shows the scattered distribution of sources in the environment, in layout way of planar presentation way. The description of such natural distribution is complex and we employed polar coordinates to specific the location or position (part b in Fig. 2). In this way of recording the location of sources in the environment, the specific location could be described as ‘{(Radius, Angle)}’. While, if there are more factors like the type, the attributes, and the scale that need to be recorded, the form could be {(Attribute, Radius, Angle, Type, Scale)}.

Polar coordinate can describe complex distributions in planar space, but it still complicates the discussion of the model and descriptions. Therefore, here we further simplify the planar spatial distribution into just four directions (part c in Fig. 2). We believe that such further simplification would greatly facilitate the focus of the discussion on key questions in this model: how to interact (choose and response) with the environment?

For this simplified compass like analysis of environment spatial pattern (part c in Fig. 2), this paper named it Cell Compass (CC) and set the recording form of source pattern as ‘SE-SS-SW-SN’ and set the source type of two possibilities as 1 (beneficial) and -1 (deleterious) in each direction. In this way, there are 16 possible distribution patterns (with in each direction, totaling 2^4^ = 16 possibilities) and they could be classified into the following three general top categories: Full, Partial, and Half (Table 2).

**Table 1:** Matrix description of the hypothesis integrating development and source-flow.

		Flow	
		With flow	Without flow
Source	Be-source	Increase	Unchanged
	De-source	Decrease	Unchanged

Type Full: this pattern refers to either all beneficial sources, named Full Be-sources (P1 in Table 2), or all deleterious sources, named Full De-sources (P6 in Table 2).

Type Half: this pattern refers to the balanced distribution of beneficial and deleterious sources. While such balance could be adjacent and continuous (P3 in Table 2), or dispersed and spaced (P4 in Table 2).

The most common situation is between types of full and half, which is either more be-source (P2 in Table 2) or more de-source (P3 in Table 2).

### Development strategy

#### Strategy 1: optimum choice of environment pattern

For more efficient development, when system face the distribution pattern of sources with six typical representative types mentioned above, there is a question we would like to ask that: which pattern would provide higher quality for development than the other patterns? Or simply which pattern is a better choice for development?

As declared in the hypothesis, for better development, system should try to obtain benefits and avoid deleterious flows. Considering the flow is generated from the source in the environment, the system choice of the environment becomes key important for system development. That means, in order to reduce investment and improve efficiency, system should choose an environment pattern where there are more be-sources than de-sources.

Considering quantitative analysis would lead to much more complex process and results in primary research stage, here in this study there’s only qualitative analysis instead of quantitative analysis of various types of source and flows. Based on such qualitative analysis, it is obvious that the pattern of P1, P2, and P3 are better than P4, P5, and P6. The optimal environment pattern is P1, and the worst environment pattern is P6.

And, the ranking of the better to worse of the six environmental patterns is: P1 > P2 > P3 > P4 > P5 > P6. While, in this ranking, P3 and P4 are almost the same, as both include two directions of be-sources and de-sources. However, considering that the be-sources in P3 are adjacent and more prone to agglomeration effects and environmental management, so we put P3 > P4.

Therefore, for more efficient development (higher benefits and lower investments), system needs to pay attention to the selecting of optimal environmental pattern. According to the discussion and ranking above, system should try to select the top three situations (P1, P2, P3), and meanwhile avoid the following three situations (P4, P5, P6).

#### Strategy 2: action and access

Facing the source and flow of the environment, system should not only choose an optimal environment (discussed in the previous section), but also adopt actions to obtain benefits and block deleterious flows to promote their own development. Therefore, in other words, the Cell model is a so called analysis pattern from ‘source’ via ‘action (or access)’ to ‘flow’ (SAF).

The possible strategies and measures could be categorized to two types: accessible or inaccessible. Take the be-source as an example, there would be two actions of accept or reject for it. While for the de-source, the two actions could be avoid or suffer. Furthermore, if the dynamic and static attributes of the source are to be considered, the measures by the system would become more complex. For example, in the face of static be-sources, system has to actively build for flow; while in the face of dynamic be-sources, system only needs to just accept it without building activity. Therefore, obviously such measures would affect the investment and cost for system development, thereby affecting their development efficiency. In order to simplify the description of system actions and related results, these sorts of specific measures would be placed in the discussion section.

**Fig. 3:**
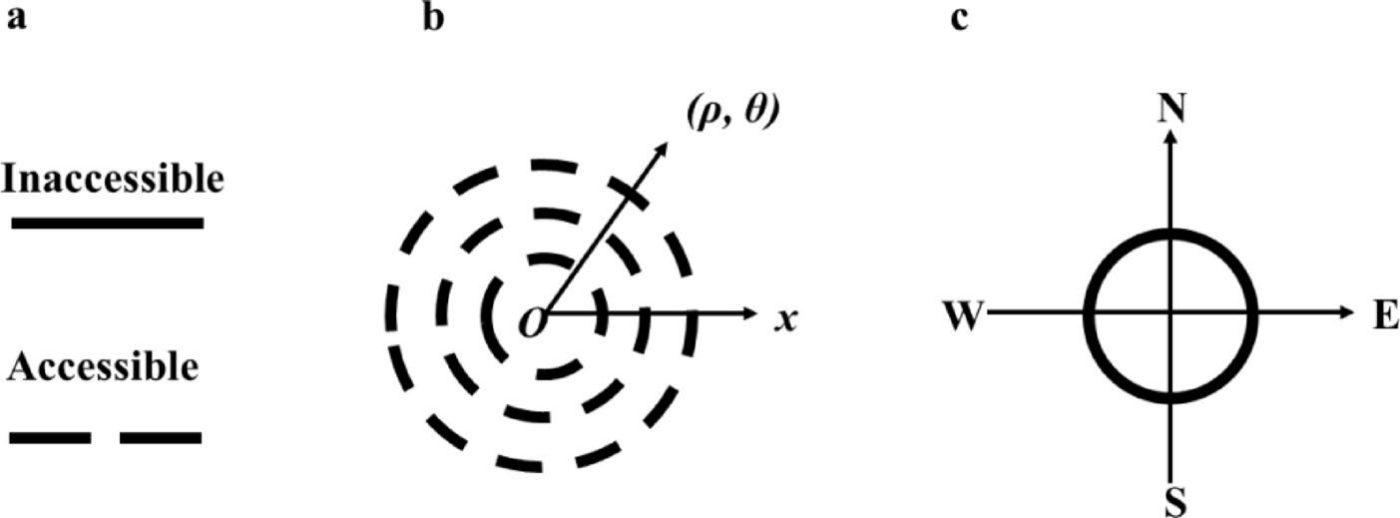
The description of action strategy (single action and spatial layout).

However, regardless of the specific actions taken by the system, the final outcome would only result in two possibilities for the flow: accessible or inaccessible, as shown in Table 1 and part a in Fig. 3.

**Table 2:**
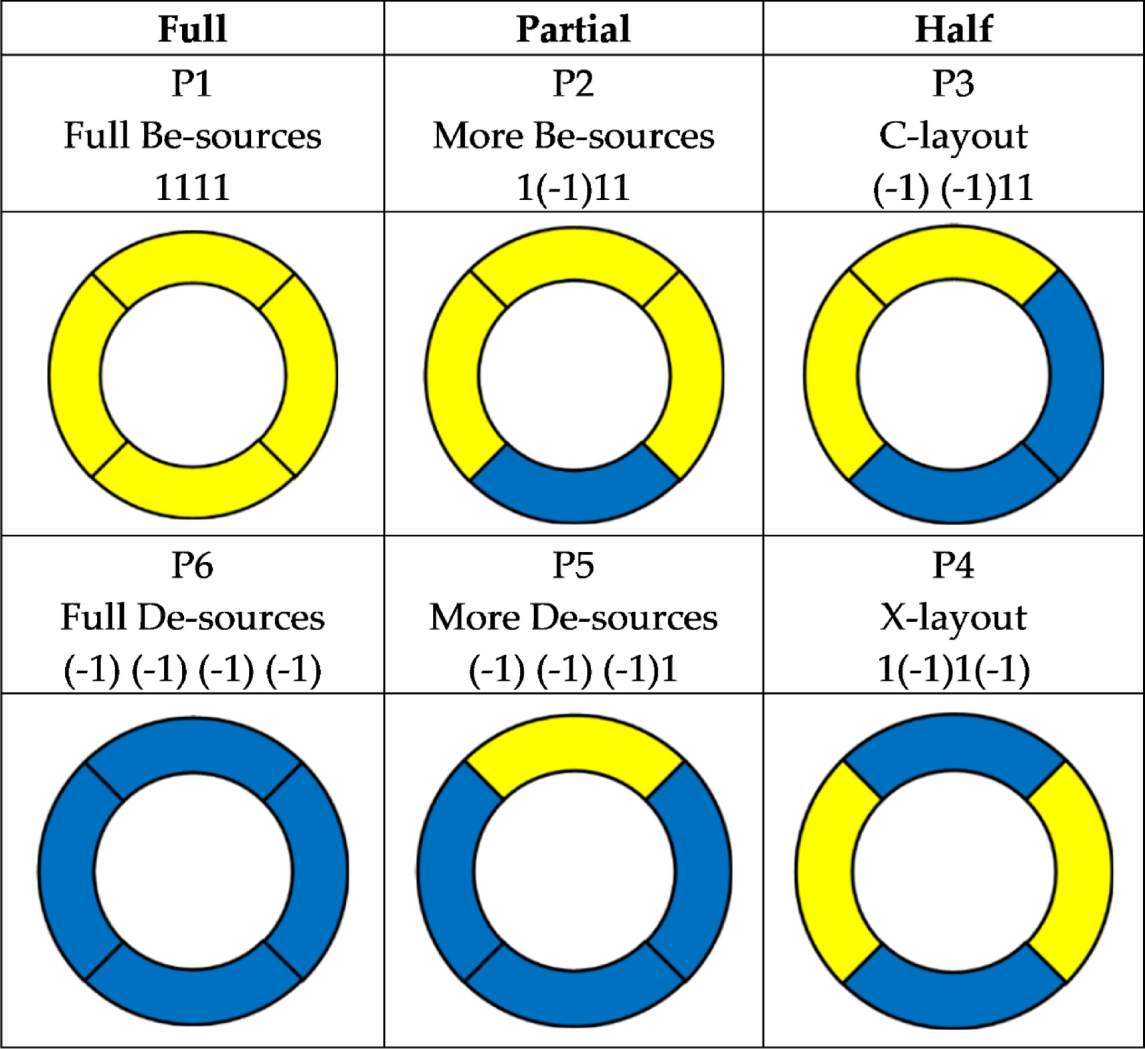
The 6 typical patterns of spatial distributions of sources.

Similar to the discussion of the complex distribution of sources in the environment, the system actions and results can also be complex, and the polar coordinates are adopted for the planar description as well. For example, when describing the accessible positions in a planar space, the result could be recorded in form of {(Radius, Angle)}, as shown in part b of Fig. 1. Further, in order to simplify the presentation of system actions and make the fundamental results and conclusions more concise, this study uses the approach of Cell Compass (CC) to describe system actions (part c in Fig. 3).

In the CC of system actions (part c in Fig. 3), the two possibilities in each direction is recorded as: ‘1’for accessible and ‘0’ for inaccessible. And, for four directions, the action distribution could be recorded as ‘AE-AS-AW-AN’ and the total 16 possibilities of the distribution could also be categorized to three top types of Full, Partial and Half, just like the situation of the sources. While, by counting of the flow number from 0 to 4, there are 6 sub-classes, as shown in the Table 3.

**Table 3:**
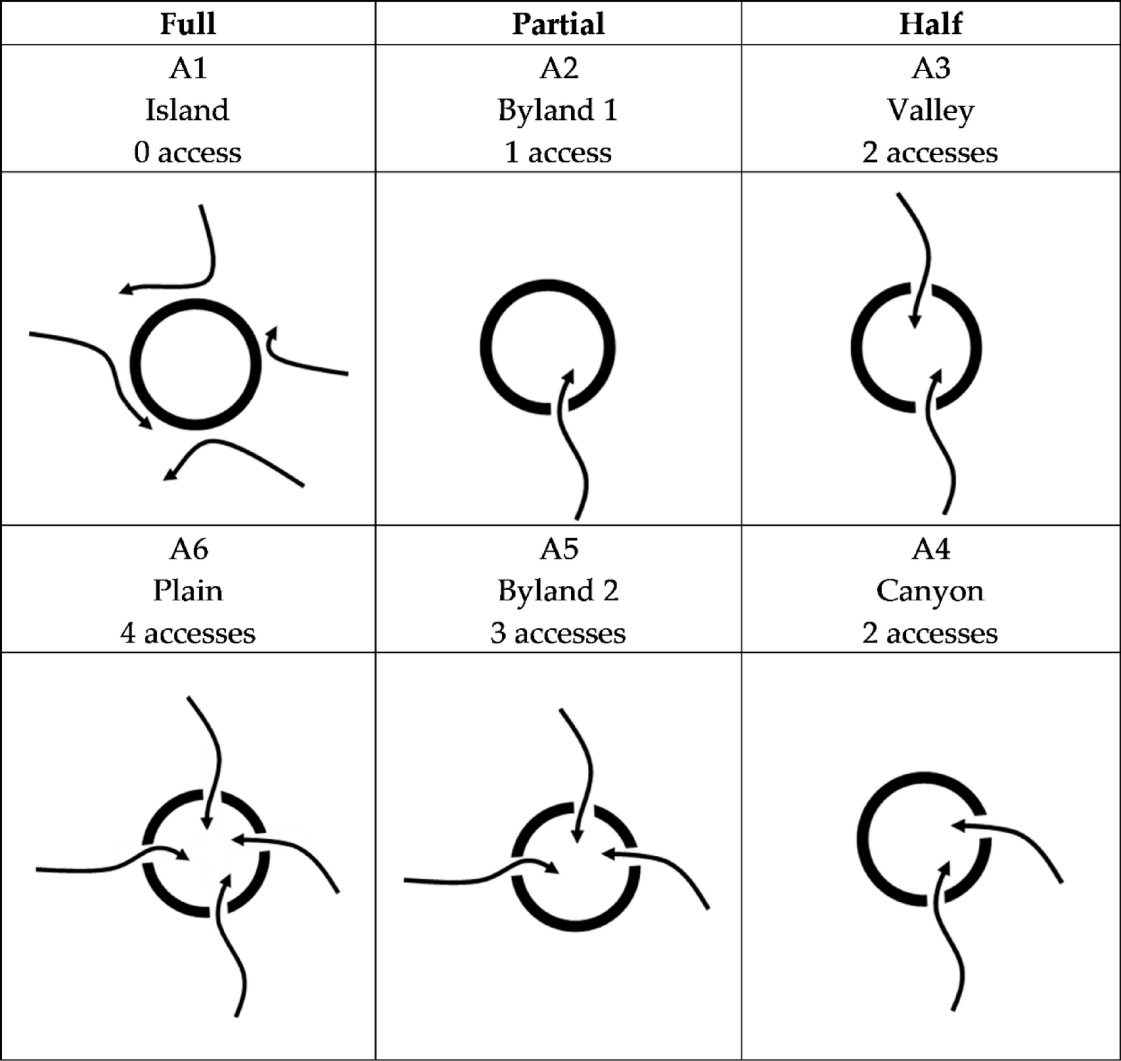
The action strategies with accesses from 0 to 4.

0 access: this type completely blocks access in all directions when facing the surrounding environment. That means, the system disconnected from the environment and seems like an island (A1 in Table 3). While, insularity situations like islands are essential scenarios in ecology researches^10,63^. The characteristic of this type is that, from a developmental perspective, if there are full of be-sources and be-flows in the environment, the system will lose many development opportunities; Conversely, from a security perspective, if there are full de-sources around, system could be self-protected quite well and avoid the danger of de-flow.

4 accesses: this type is opposite to the full reject, where each direction is available for the flows, just like the plain and ocean free for transportation (A6 in Table 3). For this type, from a development perspective, if the environmental patterns are all be-sources, it would greatly promote system development; While, from a security perspective, if the environmental patterns are all de-sources, it would greatly do harm to system development and bring significant security threats.

1 or 3 access: In these two cases, their common characteristic is that one direction action always differs from the other three directions actions, resulting in that the spatial distribution of these actions looks like a peninsula in the ocean (A2 and A5 in Table 3). From a security perspective, if all flows from three accessible directions are deleterious, then the system has to face many threats while obtaining one certain protection in the inaccessible direction (A5 in Table 3). While, the impact of this protection on system safety and development depends on its intrinsic attributes of the system. On the contrary, if there is only one direction accessible for deleterious flow, the threat to the system will be quite low, indicating that this strategy can protect the system well in a dangerous environment (A2 in Table 3).

The other two typical types of ‘Valley’ and ‘Canyon’ commonly include 2 accesses. Due to the balance of accessible and inaccessible directions, system faces situations that provide equal opportunities for both development and security.

Based on the discussion of the four typical types above, it’s obvious that for better development, the system should choose an environment pattern including four directions of be-sources and take a ‘Plain’ action; On the contrary, when facing a pattern where all four directions are de-sources, system should take the ‘Island’ strategy for security.

#### Action result: classification

Facing the environment, system would take certain action strategies and produce a result. Then we would ask: How to evaluate such results? Or which situations would be beneficial to development? The evaluation of these results needs to take counting of both environmental pattern and action strategies and this paper proposes a simple classification method for it. The following is the fundamental equation:

1 $$DT=\sum {\overrightarrow{E}}_{j}\times {\overrightarrow{I}}_{j}$$

where *DT* is the development type, $\overrightarrow{E}$_*j*_ is the type and value of environment source in location *j*, $\overrightarrow{I}$_*j*_ is the type and value of system action in location *j*.

Based on the calculation method above, the content in Table 2 could also be presented as the following Table 4:

**Table 4:** Matrix description of the hypothesis with development types.

		Flow	
		With flow: 1	Without flow: 0
Source	Be-source: 1	Increase: 1*1 = 1	Unchanged: 1*0 = 0
	De-source: (-1)	Decrease: (-1)*1 = -1	Unchanged: (-1)*0 = 0

In the CC, *j* is 4, and system would face 16 environmental patterns and also have 16 alternative action strategies. Thus, there would be 256 (16 * 16 = 256) possible results and the following is an instance of such result:

$$\begin{aligned} & {\text{Instance}}: \\ & DT\left( {\left( { - {1}} \right)\left( { - {1}} \right) \, \left( { - {1}} \right) \, \left( { - {1}} \right),{ 1111}} \right) = \left( { - {1}} \right)*{1} + \left( { - {1}} \right)*{1} + \left( { - {1}} \right)*{1} + \left( { - {1}} \right)*{1} \\ & \quad = - {4} \\ \end{aligned}$$

It is tedious to fully calculate the 256 possible results and discuss them. Just like discussing environmental patterns and countermeasures, here we only selects and discusses 6 typical situations, as shown in the following Table 5.

**Table 5:**
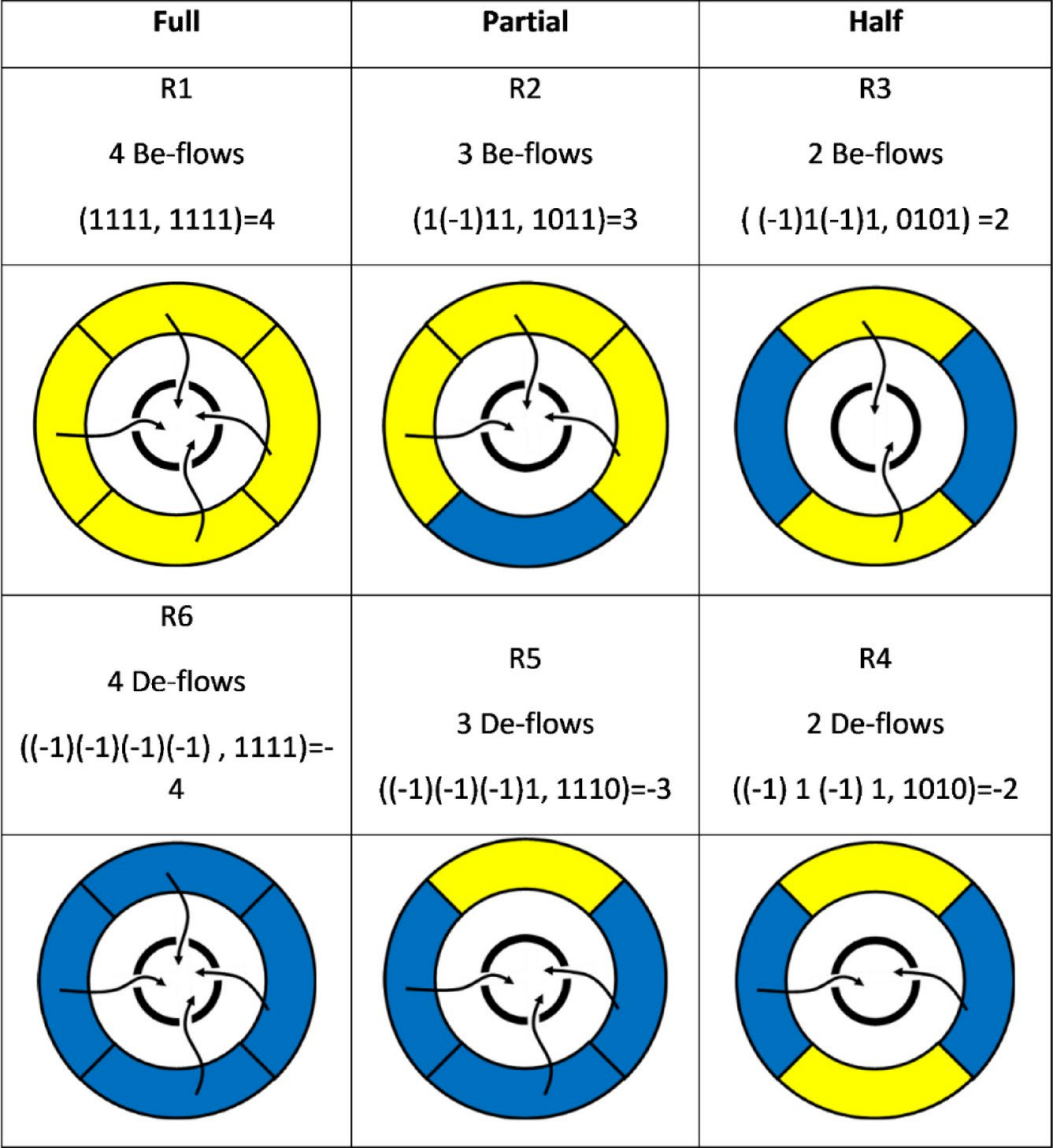
The 6 typical results based on the source pattern and action strategies.

According to the calculation method defined above, it is obvious that the three scenarios in first line are better for development, with the R1 being the best. Their development types are:

$$\begin{aligned} {\text{DT}}_{{{\text{R1}}}} & = {1}*{1} + {1}*{1} + {1}*{1} + {1}*{1} = {4}; \\ {\text{DT}}_{{{\text{R2}}}} & = {1}*{1} + \, \left( { - {1}} \right)*0 + {1}*{1} + {1}*{1} = {3}; \\ {\text{DT}}_{{{\text{R3}}}} & = \, \left( { - {1}} \right)*0 + {1}*{1} + \, \left( { - {1}} \right)*0 + {1}*{1} = {2}; \\ \end{aligned}$$

Based on the calculation result, the ranking of the three scenarios in the first line is: DT_R1_ > DT_R2_ > DT_R3._

Similarly, for the three scenarios in the second line, the development types are:

$$\begin{aligned} {\text{DP}}_{{{\text{R6}}}} & = \, \left( { - {1}} \right)*{1} + \, \left( { - {1}} \right)*{1} + \, \left( { - {1}} \right)*{1} + \, \left( { - {1}} \right)*{1} = - {4}; \\ {\text{DP}}_{{{\text{R5}}}} & = \, \left( { - {1}} \right)*{1} + \, \left( { - {1}} \right)*{1} + \, \left( { - {1}} \right)*{1} + {1}*0 = - {3}; \\ {\text{DP}}_{{{\text{R4}}}} & = \, \left( { - {1}} \right)*{1} + {1}*0 + \, \left( { - {1}} \right)*{1} + {1}*0 = - {2}; \\ \end{aligned}$$

And, the ranking of the three scenarios: DP_R4_ > DP_R5_ > DP_R6._

## Discussion

### Instance and application

The Cell model proposed in this study set up an abstract framework and related results have general implications for understanding how the environmental impacts system security and development. Additionally, like the environmental psychology, ESD and its Cell model in this study defined a broad range about the environment, encompassing natural environments, social settings, built environments, learning environments, and informational environments ^17^. It is therefore that the specific instances and scenarios contained within this abstract model could be widely present. Taking flow as an example, the typical flows include energy flow, mass flow and information flow.

Among the numerous instances, at the macro scale, taking the location selection of cities as an example, it is usually necessary to consider the natural resources available around the city, such as the flat land would be conducive to plant and harvest crops, as well as urban construction; The rivers within and around the city would be conducive to agricultural irrigation and urban domestic water use ^64,65^. Meanwhile, measures for floods should be considered for security ^66,67^; further, cities should not be too far away from transportation arteries, which are necessary for people and cargo circulation ^68,69^. The climate flow should be considered as well, like the storm, the wind, the sand, the cold wave. Therefore, it can be seen that the development of the city is closely related to the various resources and flows in the environment around ^70–72^.

In terms of specific cases analysis and applications, based on source-access-flow concepts, we once made an analysis on health security which validated how the terrain and geomorphic of the environment related with spreading flow and access affect the spreading of COVID-19 ^73^. It is actually this health security analysis that inspired the author to establish a more abstract, general, and fundamental analysis framework for both security and development issue. Interestingly, meanwhile, Tansil discussed how public green and open spaces provide conservation area for resilient and healthy besides fulfilling other human wellbeing of various social, ecological, economic, and aesthetic roles during the COVID-19 period ^74^. However, currently the framework with Cell model in this research is still at the theoretical building stage, related improvements, case study, applied research and data validations still need more following work to make this framework practical.

### Intrinsic attributes

#### Judgment of beneficial or deleterious

In the hypothesis, the judgment of the attributes of beneficial or deleterious depends on the intrinsic attributes of the system, so this judgment would be sort of subjectivity. And, this subjectivity may lead to the following differences:

1. Judgments by different system about the same source and flow would differ.
2. In different situations with new conditions, one system would make different judgments about the same source and flow.
3. The deleterious source may be transformed into beneficial source by the system.

#### Development level and efficiency

Like the definitions of ‘environmental flows (EFs)’ are “the quantity, timing, duration, frequency and quality of flows required to sustain freshwater, estuarine, and near shore ecosystems and the human livelihoods and wellbeing that depend on them” ^75^. Based on such consideration of quantity, timing, duration, frequency, this research refer to the cash flow analysis of a company to describe the system development level and efficiency, setting up an approach to quantify the effects of deleterious and beneficial flows when applying the ‘Cell model’.

When system takes actions to control the source and flow of the environment, the investment and cost will affect the development efficiency. While, how much they input depends on intrinsic attributes of the system differences. Here, to avoid the consideration trap of the complexity of intrinsic attributes and influence processes, we just only observe and measure external input–output results, so as to simplify the quantification. Based on the inputs and benefits, in a specific duration, system development level and efficiency could be described as the following:

Development level:

2 $$DL={G}_{b}-{L}_{d}-{I}_{b}-{I}_{d}$$

where *DL* is the development level, *G*_*b*_ is the gain based on the be-source, *L*_*d*_ is the loss based on the de-source, *I*_*b*_ is the investment for the be-source, *I*_*d*_ is the investment against the de-source.

Development efficiency:

3 $$DE=DL/({I}_{b}+{I}_{d})$$

where *DE* is the development efficiency, *DL* is the development level, *I*_*b*_ is the investment for the be-source, *I*_*d*_ is the investment against the de-source.

Due to differences in system attributes, the results of the same action strategy vary greatly, making it difficult to evaluate different measures without specific data. However, for one system facing the same environment, adopting different strategies would significantly affect the input and output, as well as development level and efficiency, as shown in Table 6 below.

**Table 6:** Efficiency evaluation based on different environmental sources and development strategies.

	Environment Source		Action	Result	Evaluation
S1	Be-source	Dynamic	Accept	With Be-flow	Increase without input
S2		Static	Build		Increase with input
S3		Dynamic	Prevent	Without Be-flow	Unchanged with input
S4		Static	Accept		Unchanged without input
S5	De-source	Dynamic	Accept	With De-flow	Decrease without input
S6		Static	Build		Decrease with input
S7		Dynamic	Prevent	Without De-flow	Unchanged with input
S8		Static	Accept		Unchanged without input

In Table 6, for the same final result, the action without input is always better than another action with input due to the input would make higher cost and the lower efficiency. Take scenarios of S1 and S2 for example, both of the results are increase, due to the differences of the environmental sources and corresponding actions, obviously the development efficiency of S1 is much better than S2 (S1 > S2). Similarly, for scenarios of S1 and S3, though there are same environmental source, different actions lead to different results and development efficiency (S1 > S3).

#### Development and security

In this study, many scenarios are explained from the perspective of development. However, with changes of source and flow attributes, as well as system strategies, development issues would be highly related and evolved to security issues. It is therefore that there would be different conclusions for the same situation based on different perspectives of security and development. Thus, the environmental development discussed in this paper would be sort of environmental security as well. As described in the island strategy, for an environment full of be-sources and be-flow, the development would be poor. While, for an environment full of de-sources and de-flow, the security with an island strategy would be ensured well.

## Conclusions

Security and development issues are very key topics related to human survival and sustainable development of ecosystems. There are quite many but separated theories in this area, referring to multi-disciplinary and broad discussions. This research highlighted the close relationship between the dual aspects of security and development, and reviewed separated theoretical concepts related to security and development.

Based on the review especially the theory of EEG, ES and concepts of source-flow, a unified, abstract and general ESD theory in this research is proposed to integrate the analysis of both security and development at multiple scales into one fundamental framework. A ‘Cell Model’ named ignoring the intrinsic attributes of system with its Cell compass (CC) analysis for environmental spatial pattern is also established to analyze the impact of environmental source-flow (ESF) on security and development, as well as corresponding system response strategies.

Specifically, the spatial distribution of environment patterns and system action strategies are classified and ranked due to their impact differences for security and development. Further, considering the differences of system intrinsic attributes, few possible changes and evolution as well as the calculation definition of development level and efficiency were discussed.

Up to now, this research is still at the theoretical building stage. That means this research just establishes a fundamental, integral and comprehensive framework instead of specific data or case analysis. Based on this framework, related improvements, case study, instance application and data validations should be done in the following future works. Especially, for the improvement of the theory, considering that the system would always try to optimize its actions to adjust or even control the environment to make the pattern more conducive to its own sustainable development, the relationship between a system and its environment would be a dynamic process of cyclic evolution of pattern- function-action and the analysis would become more complex.

## Data Availability

All data generated or analysed during this study are included in this published article.
